# Extirpation of the Kidney

**Published:** 1880-02

**Authors:** Herman Mynter


### Translations.

### EXTIRPATION OF THE KIDNEY.

FROM THE GERMAN BY HERMAN MYNTER, M. D.

Professor Cyerny, of Heidelberg, says, in *Centralblatt fuer Chirurgie*, that although ten years have elapsed since the first extirpation of the kidney by the celebrated Simon, we must acknowledge that the last year has done much to secure the triumph of this serious operation. The methodical use of the antiseptic treatment in ovariotomy, the antiseptic ligature and the intraperitoneal treatment of the pedicle, first made it possible for Dr. Martin, Jr., to obtain new successes in cases of movable kidneys. These operations, therefore, mark a new era in nephrotomy, because the way has been chosen through the abdominal cavity for the extirpation of the kidney, The question is no longer as to the propriety of the extirpation of the kidney, but which way is preferable, the extraperitoneal from the lumbar region, or the intraperitoneal from the linea alba. As both ways have been successful, we must determine in which case we shall choose the one, and in which the other. Martin has lately extirpated a tumor of the kidney through laparotomy with success, while Zweitel extirpated a healthy kidney with success after Simon's method, on account of a fistula between the ureter and the uterus. Czerny has tried nephrotomy twice, once without success through laparotomy on account of a large carcinoma of the kidney, the other time from the lumbar region cn account of pyonephrosis, and this was successful. The patient was a married woman, thirty-two years of age, who, for four years had experienced difficulty in urinating, until in April, 1879, an abscess formed below the right eleventh rib. The abscess was opened, and the secretion of matter was alternately more or less copious. The patient declared that, when the matter flowed freely from the fistula the urine was clear, while it became thick and purulent, and attended with great pain and fever, when for a while the fistula did not secrete anything. We were able to convince ourselves of this fact at the clinic. I diagnosticated a pyonephrosis with perinephritic suppuration on the right side, secondary catarrh at the bladder, and healthy left kidney. I tried first by dilatation of the fistula to provide for a better discharge of the matter, but held out the prospect of extirpation of the kidney, if the organ was diffusely diseased. After having, on the 22d of May, 1879, dilated the fistula in layers in the direction of the eleventh rib, and toward the middle of the crest of the ilium, my finger entered a suppurating cavity, in which I found a soft lobular tumor, which felt like placental tissue. As there occurred a copious venous bleeding, I dilated in the direction mentioned, and resected subperiostally a piece of the eleventh rib. I obtained so much space by that, that I could see how the kidney, enlarged to three times its size, was surrounded by copious old coagulas of blood. These were removed, and the posterior and lower part of the kidney loosened. As there was not sufficient space upwards, I was obliged to remove a farther piece of the eleventh rib (in all nine centimeters). I now inserted my whole hand in the wound, and loosened the upper part of the kidney, by which proceeding a large amount of matter was discharged. The pedicle was ligated with silk ligatures and elastic ligatures, the rest of the kidney was cut off except a piece of the hilus, and the ligatures brought out through the wound. The wound was disinfected with a solution of chloride of zinc (5 per cent.) and plugged with thymol-gauze, and half of the incision (20 centimeters long) sewed together. Scarcely any fever occurred. On the 14th of June the necrotic pedicle and the ligatures came away. On the 16th of June the patient got up, but was not discharged until July 3d, as the wound closed very slowly. Sept. 14th, there was still a slight discharge from the wound, but the patient feels perfectly well, and is said to be pregnant. I have no doubt that both methods of nephrotomy, the extraperitoneal and the intraperitoneal, are justifiable. I farther believe that the extraperitoneal method, ceteris paribus, is the less serious. If the kidney is not too much enlarged and fixed, the lumbar nephrotomy is preferable. For movable kidney on the other side laparotomy ought to be chosen. Whether laparotomy may also be of use in fixed tumors of the kidney, which are too large for the lumbar operation, will be learned by bold trials in such cases in the future. I believe I have enlarged the field for the lumbar operation by making the incision of Simon farther forward, at the anterior margin of the quadratus lumborum muscle, and by combining it with the partial resection of the eleventh rib. The whole hand may then be introduced with ease into the wound, and the loosening of the kidney and the ligature of the pedicle may be accomplished with greater facility. The resection of the rib is without danger, because the outer third of the eleventh rib has no connection with the pleural cavity.

